# Verminous Ophthalmia (Intra-Ocular Filariosis)

**Published:** 1900-03

**Authors:** A. G. Hopkins

**Affiliations:** University of Wisconsin, Madison, Wis.


### VERMINOUS OPHTHALMIA (INTRA-OCULAR FILARIOSIS).

By A. G. Hopkins, V.S.,
UNIVERSITY OF WISCONSIN, MADISON, WIS.

During a course of practice in Manitoba I was fortunate enough to run across a case of the above-mentioned disease. The owner had consulted me about the case some three weeks previous to allowing me to make a clinical examination and diagnosis. His description of the case led me to believe that I had a case of simple ophthalmia, as he naively put it ; a scum was growing over the eye, which burnt alum and powdered glass could not remove. I strongly deprecated such heroic measures, and insisted on a clinical examination. The animal (a mare) was brought to me showing profuse lachrymation, photophobia, and conjunctivitis. In spite of the partial opacity of the cornea, the filaria could be readily seen performing rapid evolutions in the fluid of the anterior chamber. An operation being decided upon, the animal was placed in a recumbent position, the eye was cocained (5 per cent. solution) and the technique of Williams (*Veterinary Surgery*) adopted. I was indebted to a medical confrere for a scalpel with which to make the incision at the upper and outer edge of the cornea. The scalpel was introduced at an angle so as to make a valvular incision. A fine pair of forceps were then used to dilate the incision when the filaria was somewhat forcibly expelled. Little of the humor (aqueous) escaped, but the parasite died on reaching the extra-ocular position, whether due to contact with some of the anæsthetic or not I am unable to say. The eye was treated with cold boric acid compresses, and later with a mild atropia-zinc sulphate lotion. I did not see the patient again. The owner, however, one month later reported the opacity of the cornea as disappearing, and stated that the mare could see quite well and that the intolerance of light had disappeared. The filaria was about 2.5 inches long, thin and white, with a smooth integument, and is now resting in glycerin. I consider the rationale of Williams' technique for the keratotomy preferable to that given by Neumann.

---

Dr. Hugh Thomson, of Shabbona, Ill., reports a fatal result from overdose of arecoline hydrobromata. Subject: a four-year-old gelding, weighing over 1600 pounds. Two grains of the drug were injected in the chest muscles, followed in five minutes by profuse salivation; in twenty-five minutes by passage of feces. The animal was down when medicine was given, but rose up at the first movement of bowels; pain seemed to have left him. Several movements of the bowels followed during the first hour, becoming liquid in character, and, though the pain ceased, the liquid stools continued for some thirty hours, at which time he died.

On autopsy the stomach and bowels were inflamed and contained a vast amount of a bloody flux, and death seemed to have followed from the gastro-enteritis set up and superpurgation produced.

---

The presence of rabies among sheep in Western Pennsylvania is said to have caused the loss of some seventy-five head in one flock.

Missouri, Kansas, and Texas livestock sanitary boards require that all cattle shipped into those States for breeding purposes shall be accompanied by official certificates of health, including the tuberculin-test. Many such cattle shipped through Kansas City are examined by government veterinary inspectors.

The Holstein-Freisian Association is determined to force sister associations of milk-cattle to a test of the relative merits of the several breeds as to butter-making.

